# Characterization of the major histocompatibility complex locus association with Behçet’s disease in Iran

**DOI:** 10.1186/s13075-015-0585-6

**Published:** 2015-03-19

**Authors:** Joana M Xavier, Fereydoun Davatchi, Olga Abade, Farhad Shahram, Vânia Francisco, Bahar Sadeghi Abdollahi, Hélder Trindade, Abdolhadi Nadji, Niloofar Mojarad Shafiee, Fahmida Ghaderibarmi, Dário Ligeiro, Sofia A Oliveira

**Affiliations:** Instituto de Medicina Molecular, Faculdade de Medicina, Universidade de Lisboa, Avenida Professor Egas Moniz, Edifício Egas Moniz, 1649-028 Lisboa, Portugal; Instituto Gulbenkian de Ciência, Oeiras, Portugal; Rheumatology Research Center, Tehran University of Medical Sciences, Tehran, Iran; Lisbon Center for Blood and Transplantation, Instituto Português de Sangue e Transplantação, Lisboa, IP Portugal

## Abstract

**Introduction:**

The aim of this study was to characterize the association of human leukocyte antigen (HLA) *B* alleles and major histocompatibility complex (MHC) single nucleotide polymorphisms (SNPs) with Behçet’s disease (BD) in an Iranian dataset.

**Methods:**

The association of three SNPs in the MHC region previously identified as the most associated in high-density genotyping studies was tested in a case–control study on 973 BD patients and 825 controls from Iran, and the association of *HLA-B* alleles was tested in a subset of 681 patients and 414 controls.

**Results:**

We found that HLA-B*51 (*P* = 4.11 × 10^−41^, OR [95% CI] = 4.63[3.66-5.85]) and B*15 confer risk for BD (*P* = 2.83 × 10^−2^, OR [95% CI] = 1.75[1.08-2.84]) in Iranian, and in B*51 negative individuals, only the B*15 allele is significantly associated with BD (*P* = 2.51 × 10^−3^, OR [95% CI] = 2.40[1.37-4.20]). rs76546355, formerly known as rs116799036, located between *HLA-B* and *MICA* (MHC class I polypeptide-related sequence A), demonstrated the same level of association with BD as HLA-B*51 (*P*_adj_ = 1.78 × 10^−46^, OR [95% CI] = 5.46[4.21-7.09], and *P*_adj_ = 8.34 × 10^−48^, OR [95% CI] = 5.44[4.20-7.05], respectively) in the *HLA-B* allelotyped subset, while rs2848713 was less associated (*P*_adj_ = 7.14 × 10^−35^, OR [95% CI] = 3.73[2.97-4.69]) and rs9260997 was not associated (*P*_adj_ = 1.00 × 10^−1^). Additionally, we found that B*51 genotype-phenotype correlations do not survive Bonferroni correction, while carriers of the rs76546355 risk allele predominate in BD cases with genital ulcers, positive pathergy test and positive BD family history (2.31 × 10^−4^ ≤ *P* ≤ 1.59 × 10^−3^).

**Conclusions:**

We found that the HLA-B*51 allele and the rs76546355/rs116799036 MHC SNP are independent genetic risk factors for BD in Iranian, and that positivity for the rs76546355/rs116799036 risk allele, but not for B*51, does correlate with specific demographic characteristics or clinical manifestations in BD patients.

**Electronic supplementary material:**

The online version of this article (doi:10.1186/s13075-015-0585-6) contains supplementary material, which is available to authorized users.

## Introduction

Behçet’s disease (BD) is a systemic immuno-inflammatory disorder classified as a vasculitis and affects blood vessels of nearly all sizes and types, especially in the mucocutaneous and ocular structures. The most common manifestations are recurrent orogenital ulcerations and ophthalmologic inflammatory lesions, but may also involve the vascular, neurological, articular, respiratory, and gastrointestinal systems. The etiopathology of BD remains uncertain, but infectious or environmental factors are thought to trigger an excessive immune and/or inflammatory response in genetically predisposed individuals.

The human leukocyte antigen class I allele HLA-B*51 is a well-established and universal genetic risk factor for BD, but the susceptibility conferred by other HLA-B alleles such as B*15 or B*35 remains controversial [1,2]. Additionally, recent genome-wide association studies (GWAS) and in-depth investigations of the major histocompatibility complex (MHC) region have suggested independent associations of other HLA alleles (for example, HLA-A*26, HLA-Cw*1602) and of other SNPs or genes located in the MHC locus (for example, *PSORS1C1*) [3-7]. However, these reports have also been inconsistent, have not been validated by independent research groups, and have not systematically addressed whether an SNP or an HLA allele is the strongest risk factor for BD. Furthermore, numerous small studies have investigated genotype-phenotype correlations in BD patients (reviewed and meta-analyzed in Maldini *et al*. [8]), but well-powered studies exploring the association of risk genotypes with all the major clinical and demographic characteristics of BD cases are lacking.

In this context, the aim of this study was to better characterize the risk conferred by HLA-B alleles and MHC SNPs for Behçet’s disease in an Iranian dataset and to test if there is a correlation between the genotypes at the strongest genetic risk factors and characteristics of BD patients.

## Methods

### Study subjects

The study participants were enrolled in the Behçet’s disease outpatient clinic, Rheumatology Research Center, Shariati Hospital, at the Tehran University of Medical Sciences, Tehran, Iran from 2007 to 2012. The diagnosis of BD was made according to the revised International Criteria for Behçet’s Disease [9]. Patients with age at onset of BD after 60 years were excluded. Controls were evaluated using the same evaluation procedures as the cases and selected when negative for BD, any other rheumatological or auto-immune disorder and oral aphthosis. The clinical and demographic features of the participants were obtained by medical interview at the time of blood sampling and inspection of medical records. The clinical characteristics described in Table 1 are defined as follows: skin lesions include pseudo folliculitis, erythema nodosum, and skin aphthosis; ophthalmologic manifestations refer to anterior uveitis, posterior uveitis, and retinal vasculitis; joint manifestations include arthralgia, arthritis, and ankylosing spondylitis; neurological manifestations include peripheral and central manifestations; vascular involvement refers to arterial thrombosis, large vein thrombosis, phlebitis, and superficial phlebitis; gastrointestinal manifestations include chronic diarrhea and proctorrhagia. Even though approximately 9% of the cases and controls have a family history of BD, all cases and controls are not known to be genetically related.

**Table 1:** **Clinical and demographic characterization of the dataset**

**Characteristic**	**Total dataset**		**HLA-B allelotyped subset**	
	**Cases**	**Controls**	**Cases**	**Controls**
**Number**	973	825	681	414
**Male gender, n/total n (%)**	511/973 (52.5)	348/825 (42.2)	480/681 (70.5)	281/414 (67.9)
**Age at examination, years, mean ± SD**	39.1 ± 10.9	40.5 ± 11.9	39.2 ± 10.6	42.7 ± 11.5
**Age at onset, years, mean ± SD**	26.0 ± 9.6		26.0 ± 9.5	
**Oral aphthosis, n/total n (%)**	962/973 (98.9)	0/825 (0.0)	672/681 (98.7)	0/414 (0.0)
**Genital aphthosis, n/total n (%)**	609/973 (62.6)	0/825 (0.0)	424/681 (62.3)	0/414 (0.0)
**Skin lesions, n/total n (%)**	539/973 (55.4)		393/681 (57.7)	
**Ophthalmologic manifestations, n/total n (%)**	583/973 (59.9)		427/681 (62.7)	
**Joint manifestations, n/total n (%)**	300/973 (30.8)		218/681 (32.0)	
**Neurological manifestations, n/total n (%)**	61/973 (6.3)		42/681 (6.2)	
**Vascular involvement, n/total n (%))**	51/973 (5.2)		43/681 (6.3)	
**Gastrointestinal manifestations, n/total n (%)**	38/973 (3.9)		22/681 (3.2)	
**Pathergy phenomenon, n/total n (%)**	441/959 (46.0)		323/669 (48.3)	
**Family history of BD, n/total n (%)**	85/935 (9.1)		57/651 (8.8)	

n/total n = number of individuals/total number of cases or controls analysed.

This study received ethical approval from the ethics committee at the Tehran University for Medical Sciences, Iran. All participants were informed of the study, provided informed written consent and the study was conducted according to the Declaration of Helsinki.

### HLA-B class I typing

Blood samples were drawn into EDTA tubes and stored at −20°C until genomic DNA was extracted using QIAamp® DNA Blood Maxi kits (QIAGEN, Hilden, Germany) or a salting out procedure and diluted in Tris-EDTA buffer. The concentrations of extracted DNA were determined by Nanodrop using absorbance readings at 260 nm and the DNA was then stored at 4°C.

A random subset (681 BD cases and 414 controls) of the total dataset was typed for HLA-B with a hybridization bead-based array using reverse-sequence specific oligo-probes (RSSOP) detected with a Luminex X-100 (Luminex Corp., Austin, TX, USA). The assays were performed at the Lisbon Center for Blood and Transplantation (Lisboa, Portugal) with lot#11 of LABType RSSO1B reagents (One Lambda, Canoga Park, CA, USA) according to manufacturer instructions.

### Genotyping

Individual samples were genotyped in the Genomics Unit of the *Instituto Gulbenkian de Ciência* (Oeiras, Portugal) using the Sequenom iPlex assay (San Diego, CA, USA), according to the manufacturer’s protocol, and detected in a Sequenom MassArray K2 platform. The primer sequences (Additional file 1: Table S1) were designed using the Sequenom MassArray Assay Design V.3.0 software. Extensive quality control was performed using eight HapMap [10] controls of diverse ethnic affiliations, sample duplication within and across plates, Mendelian inheritance check in three large pedigrees (used for quality control purposes only), Hardy-Weinberg equilibrium (HWE) in the control group (*P* >0.001), and a call rate for each SNP >90%. Genotype determinations were performed blinded to affection status (control or case). Individual DNA samples with genotyping success rate across all SNPs <85% were also excluded.

### Association analysis

The unpaired Student’s *t*-test and the chi-square (*χ*2) test was used to compare quantitative and qualitative clinical and demographic data between BD cases and controls, respectively. SNP association analysis, unadjusted and adjusted for gender and pairwise conditional analysis, was performed with logistic regression using the SNPassoc V.1.4-9 package [11] implemented in R [12]. The *χ*2 tests were performed to explore the association of each HLA-B allele with BD (each allele was compared with all other alleles pooled together) and to test the association of HLA-B*51 and the A allele of rs76546355/rs116799036 to BD cases clinical features. The *χ*2 test for Hardy-Weinberg equilibrium in controls and linkage disequilibrium (LD) calculation was performed using Haploview 4.2 [13]. The odds ratio (OR) and associated 95% CI was calculated for SNPs with significant associations with BD. Nominal significance (*P* ≤5.00 × 10^−2^) was used to declare an association significant in the association analysis of the HLA-B alleles with BD (as the tests performed are not independent), and in the association of SNPs with BD (as these constitute replications of previously reported associations). For the association of clinical manifestations in BD patients with genetic variants, results were declared significant below the Bonferroni correction level (*P* ≤4.17 × 10^−3^ for 12 tested characteristics).

## Results

### Dataset characterization

The main clinical and demographic characteristics of the 973 BD cases and 824 controls used in the SNP association study are summarized in Table 1. The distribution of clinical symptoms in the Iranian BD patients (for example, 98.9% with oral aphthosis, 62.6% with genital aphthosis, 59.9% with ophthalmological manifestations) is in line with observations for larger datasets [9,14], but skin lesions (55.4%) and gastrointestinal manifestations (3.9%) are less frequent than previously reported in Iranians (64.9% to 73.6% and 7.4% to 8.8%, respectively) [15-17]. The average age at examination (AAE) was slightly higher in controls when compared to BD cases (mean AAE ± SD of 39.1 ± 10.9 years in BD and 40.5 ± 11.9 years in controls, *P* = 1.16 × 10^−2^) but this nominally significant difference did not withstand Bonferroni correction for all the clinical variables tested. Male-to-female ratio was increased in BD patients (*P* = 1.23 × 10^−5^). As the percentage of males has been reported to influence the strength of association between HLA-B*51 and BD [18] and may also influence the association of genetic variants in the *HLA-B* region, gender was included as a covariate in adjusted logistic regression models of association.

BD cases (n = 681) and controls (n = 414) randomly selected from our total BD dataset were allelotyped for *HLA-B*. The characteristics of this smaller group of individuals are similar to those of the full dataset with the exception of the male gender, which is more prevalent in the *HLA-B* allelotyped dataset (Table 1).

### Association of *HLA-B* alleles with BD risk

To investigate the association of *HLA-B* alleles with BD in the Iranian population, we first allelotyped *HLA-B* in 681 BD cases and 414 controls. Among the 31 observed *HLA-B* alleles, 14 had frequencies ≤2% in controls (alleles B*58, B*37, B*39, B*57, B*53, B*42, B*73, B*45, B*47, B*56, B*46, B*48, B*54, B*67) and were pooled together in Table 2. The association of the remaining 17 *HLA-B* alleles with BD susceptibility was then assessed individually by testing each allele against all others pooled together.

**Table 2:** **HLA-B allele frequencies and their association with Behçet’s disease (BD)**

	**HLA-B allelotyped dataset**				HLA-B*51 negative individuals			
**HLA-B allele**	**Cases**	**Controls**	***P*** **-values**	**Odds ratio**	**Cases**	**Controls**	***P*** **-values**	**Odds ratio**
				**(95% CI)**				**(95% CI)**
B*35	13.2%	22.0%	**1.19 × 10** ^**−7**^	0.54 (0.43, 0.68)	22.2%	25.3%	2.72 × 10^−1^	
B*51	39.1%	12.1%	**4.11 × 10** ^**−41**^	4.63 (3.66, 5.85)	-	-	-	
B*52	3.0%	5.8%	**1.99 × 10** ^**−3**^	0.50 (0.33, 0.77)	5.3%	6.5%	4.72 × 10^−1^	
B*18	4.2%	5.7%	1.37 × 10^−1^		7.5%	6.7%	6.90 × 10^−1^	
B*07	2.8%	5.0%	**1.20** × **10** ^**−2**^	0.55 (0.35, 0.86)	4.2%	5.6%	3.62 × 10^−1^	
B*13	2.2%	4.1%	**1.49** × **10** ^**−2**^	0.53 (0.32, 0.87)	3.7%	4.7%	5.58 × 10^−1^	
B*08	2.5%	4.0%	6.66 × 10^−2^		3.3%	4.2%	5.51 × 10^−1^	
B*38	3.3%	3.9%	5.68 × 10^−1^		7.0%	4.8%	1.51 × 10^−1^	
B*55	3.7%	3.9%	9.08 × 10^−1^		5.3%	4.3%	5.64 × 10^−1^	
B*40	1.6%	3.7%	**2.70 × 10** ^**−3**^	0.42 (0.24, 0.73)	2.2%	4.0%	1.31 × 10^−1^	
B*44	3.4%	3.7%	8.10 × 10^−1^		5.9%	4.2%	2.37 × 10^−1^	
B*41	2.3%	3.5%	1.16 × 10^−1^		2.9%	4.2%	3.20 × 10^−1^	
B*50	2.6%	3.5%	2.60 × 10^−1^		5.1%	3.7%	3.53 × 10^−1^	
B*14	2.0%	3.1%	1.17 × 10^−1^		2.9%	3.6%	6.34 × 10^−1^	
B*49	1.5%	3.0%	**2.89 × 10** ^**−2**^	0.50 (0.28, 0.90)	1.8%	3.4%	1.42 × 10^−1^	
B*15	4.8%	2.8%	**2.83 × 10** ^**−2**^	1.75 (1.08, 2.84)	7.5%	3.3%	**2.51 × 10** ^**−3**^	2.40 (1.37, 4.20)
B*27	2.6%	2.5%	9.27 × 10^−1^		4.8%	2.8%	1.05 × 10^−1^	
**Other**	5.2%	7.6%			8.4%	8.7%		

The complete dataset allelotyped for human leukocyte antigen (HLA)-B includes 681 BD cases and 414 controls, and the subset of HLA-B*51-negative individuals is composed of 227 BD cases and 323 controls. Other (first column) includes alleles with a frequency ≤2.0% in the control group. Nominally significant *P*-values are highlighted in bold and the respective odds ratio and 95% CI are indicated.

The HLA-B*51 genotype distribution (and frequencies) were the following: 79 (11.6%) patients were homozygotes (B*51/B*51), 375 (55.1%) were heterozygotes (B*51/any other HLA-B allele), and 227 (33.3%) did not have any B*51 allele; 9 (2.2%) controls were homozygous for B*51, 82 (19.8%) were heterozygous for B*51, and 323 (78.0%) were not carriers of B*51. Therefore, in our dataset, the B*51 allele frequency was 39.1% in cases and 12.1% in controls (Table 2), and 66.7% of cases and 22.0% of controls were B*51-positive (B*51/B*51 homozygotes plus B*51 heterozygotes). As expected, the HLA-B*51 allele significantly increased the BD risk (*P* = 4.11 × 10^−41^, OR (95% CI) = 4.63 (3.66, 5.85)). Even though to a lesser degree, HLA-B*15 also appeared to confer risk for BD (*P* = 2.83 × 10^−2^, OR (95% CI) = 1.75 (1.08 to 2.84)). Conversely, the B*35, B*52, B*07, B*13, B*40, and B*49 alleles seemed to be protective (1.19 × 10^−7^ ≤ *P* ≤2.89 × 10^−2^) (Table 2).

AS the strong difference in HLA-B*51 allele frequencies between cases and controls may secondarily and artificially affect or mask the association of the other alleles, we performed case-control association tests in the subset of samples negative for B*51. We tested their association in the 227 BD cases (33.3%) and 323 controls (78.0%) who were not carriers of the B*51 allele. In this group of individuals, only the B*15 allele remained significantly associated (*P* = 2.51 × 10^−3^, OR (95% CI) = 2.40 (1.37, 4.20)) with BD (Table 2).

### Association of MHC SNPs with BD risk

To confirm the previously described associations with BD of polymorphisms in the MHC locus and to compare them with the HLA-B*51 association in an Iranian sample, we selected the top four MHC SNPs identified in GWAS conducted on Japanese, Chinese and Turkish samples (rs4959053 from Mizuki *et al*. [19] and Hou *et al*. [5], rs9260997 and rs2848713 from Remmers *et al*. [4], and rs4947296 from Lee *et al*. [6]) and the top SNP (rs116799036, currently known as rs76546355) identified on a recent and exhaustive study of the MHC locus in Italian and Turkish BD cases and controls [7]. The rs4947296 SNP failed in the assay design phase and the rs4959053 SNP did not pass the HWE quality control check (*P* = 8.00 × 10^−4^ in the control samples), and therefore their association with BD was not assessed.

The association results of these three polymorphisms are shown in Table 3. rs9260997 in the *HLA-G/H/J* gene was nominally associated with BD in the full dataset (*P*_adj_ = 2.20 × 10^−2^, OR (95% CI) = 1.31 (1.04, 1.67)) but not in the subset allelotyped for HLA-B (*P*_adj_ = 1.00 × 10^−1^). rs76546355/rs116799036, located between *HLA-B* and *MICA*, and rs2848713, located downstream of *MICA*, were both strongly associated with BD in the full dataset (*P*_adj_ = 9.34 × 10^−68^, OR (95% CI) = 4.56 (3.78 to 5.50), and *P*_adj_ = 4.90 × 10^−55^, OR (95% CI) = 3.52 (2.97, 4.18), respectively). In the HLA-B typed subset, the association of rs76546355/rs116799036 (*P*_adj_ = 1.78 × 10^−46^, OR (95% CI) = 5.46 (4.21, 7.09)) was similar to that of HLA-B*51 (*P*_adj_ = 8.34 × 10^−48^, OR (95% CI) = 5.44 (4.20, 7.05)) and stronger than that of rs2848713 (*P*_adj_ = 7.14 × 10^−35^, OR (95% CI) = 3.73 (2.97, 4.69)).

**Table 3:** **Association between MHC single nucleotide polymorphisms (SNP) and Behçet’s disease in the full dataset and on the subset allelotyped for HLA-B**

**Dataset**	**dbSNP ID**	**Gene or**	**Allele**	**Frequency**		***P***_**unadj**_ **-values**	***P***_**adj**_ **-values**	**Odds ratio (95% CI)**
		**nearest gene**		**Cases**	**Controls**			
**Total**	rs9260997	*HLA-G/H/J*	C	0.911	0.886	**2.03 × 10** ^**−2**^	**2.20 × 10** ^**−2**^	1.31 (1.04, 1.67)
	rs76546355	*HLA, B - MICA*	A	0.388	0.140	**2.51 × 10** ^**−69**^	**9.34 × 10** ^**−68**^	4.56 (3.78, 5.50)
	rs2848713	*MICA*	A	0.422	0.184	**2.12 × 10** ^**−56**^	**4.90 × 10** ^**−55**^	3.52 (2.97, 4.18)
**HLA-B allelotyped**	rs9260997	*HLA-G/H/J*	C	0.908	0.886	1.06 × 10^−1^	1.00 × 10^−1^	
	rs76546355	*HLA-B - MICA*	A	0.388	0.126	**9.84 × 10** ^**−47**^	**1.78 × 10** ^**−46**^	5.46 (4.21, 7.09)
	rs2848713	*MICA*	A	0.427	0.184	**4.83 × 10** ^**−35**^	**7.14 × 10** ^**−35**^	3.73 (2.97, 4.69)
		*HLA-B*	B*51	0.391	0.121	**5.86 × 10** ^**−48**^	**8.34 × 10** ^**−48**^	5.44 (4.20, 7.05)

For comparison, the association of the human leukocyte antigen (HLA)-B*51 allele using a log-additive model of association, unadjusted (*P*_unadj_) and adjusted (*P*_adj_) for gender, is shown at the bottom of the table. Nominally significant *P*-values are highlighted in bold and the respective odds ratios and 95% CI are indicated for the adjusted model.

To determine if the associations of rs76546355/rs116799036, rs2848713, and HLA-B*51 are independent, we performed pairwise conditional logistic regression analyses (Table 4). Except for the association of rs2848713 conditioned on rs76546355/rs116799036, which was abrogated (*P*_adj_ = 9.39 × 10^−1^), all pairwise conditional associations remained significant even though the strength of the association decreased substantially (from 7.14 × 10^−35^ ≤ *P*_adj_ ≤8.34 × 10^−48^ to 1.84 × 10^−17^ ≤ *P*_adj_ ≤5.42 × 10^−4^). These results are consistent with the low pairwise LD observed in the controls between these two SNPs and the HLA-B*51 allele (*r*^2^ ≤ 0.59) (Table 4).

**Table 4:** Pairwise linkage disequilibrium (LD) and conditional association results for rs76546355, rs2848713, and HLA-B*51

	**Covariate**	**rs76546355**		**rs2848713**		HLA-B*51	
**Variant**		***r*** ^**2**^	***P***_**adj**_ **-values, OR (95% CI)**	***r*** ^**2**^	***P***_**adj**_ **-values, OR (95% CI)**	***r*** ^**2**^	***P***_**adj**_ **-values, OR (95% CI)**
**rs76546355**		-		0.59	**5.63 × 10** ^**−14**^, 5.56 (3.52, 8.79)	0.58	**1.24 × 10** ^**−7**^, 2.54 (1.79, 3.61)
**rs2848713**			9.39 × 10^−1^	-		0.35	**5.42 × 10** ^**−4**^, 1.68 (1.25, 2.26)
HLA-B*51			**1.78 × 10** ^**−8**^, 2.75 (1.93, 3.92)		**1.84 × 10** ^**−17**^, 3.92 (2.83, 5.44)	-	

LD values were computed in the control samples only. Nominally significant *P*-values are highlighted in bold and the respective odds ratio (OR) and 95% CI are indicated. *P*-values were adjusted for gender and calculated in the subset of individuals allelotyped for human leukocyte antigen (HLA)-B.

### Phenotype-genotype relationships in Iranian BD patients

To investigate if the HLA-B*51 or the rs76546355/rs116799036 risk allele status are associated with clinical and demographic characteristics of Iranian BD patients, we tested whether the characteristics have significantly different frequencies among B*51-positive (66.7% or 454 out of 681 patients) and B*51-negative cases (33.3% or 227 out of 681 patients), or between (AA + GA) patients when compared to GG patients (Table 5). The oral aphthosis variable was not studied because this clinical phenotype is present in virtually all BD patients. We found that B*51 carriers have a higher risk of a positive pathergy test reaction (*P* = 7.34 × 10^−3^, OR (95% CI) = 1.56 (1.13, 2.16)) and of a positive family history of BD (*P* = 2.35 × 10^−2^, OR (95% CI) = 2.16 (1.09, 4.27)), but these associations were not significant after Bonferroni correction. Positivity for the risk allele at rs76546355/rs116799036 (A) predominates in BD cases with genital ulcers (*P* = 1.05 × 10^−3^, OR (95% CI) = 1.59 (1.20, 2.09)), positive pathergy test (*P* = 2.31 × 10^−4^, OR (95% CI) = 1.69 (1.28, 2.23)) and positive BD family history (*P* = 1.59 × 10^−3^, OR (95% CI) = 2.52(1.40, 4.58), and these associations withstand Bonferroni correction.

**Table 5:** Association of Behçet’s disease (BD) characteristics with human leukocyte antigen (HLA)-B*51 and rs76546355

	HLA-B*51				**rs76546355**			
**Characteristic**	B*51 ^**+**^	B*51 ^**−**^	***P*** **-values**	**OR (95% CI)**	**AA + GA**	**GG**	***P*** **-values**	**OR (95% CI)**
**Male sex, %**	72.2	67.0	1.54 × 10^−1^		54.4	48.6	9.07 × 10^−2^	
**Age at onset, years, mean ± SD**	26.3 ± 9.1	25.5 ± 10.2	3.02 × 10^−1^		26.0 ± 9.1	25.8 ± 10.4	7.25 × 10^−1^	
**Genital aphthosis, %**	62.8	61.2	6.96 × 10^−1^		66.5	55.6	**1.05 × 10** ^**−3**^	1.59[1.20-2.09]
**Skin lesions, %**	59.7	53.7	1.39 × 10^−1^		56.7	52.7	2.38 × 10^−1^	
**Ophthalmologic manifestations, %**	63.0	62.1	8.23 × 10^−1^		59.2	61.6	4.88 × 10^−1^	
**Joint manifestations, %**	33.9	28.2	1.31 × 10^−1^		32.0	28.9	3.32 × 10^−1^	
**Neurological manifestations, %**	5.9	6.6	2.03 × 10^−1^		5.6	4.4	8.85 × 10^−1^	
**Vascular involvement, %**	5.3	7.5	7.36 × 10^−1^		6.3	6.0	4.35 × 10^−1^	
**Gastrointestinal manifestations, %**	3.3	3.1	8.79 × 10^−1^		3.6	4.4	5.28 × 10^−1^	
**Positive pathergy test, %**	51.8	40.8	7.34 × 10^−3^	1.56 (1.13, 2.16)	50.2	37.5	**2.31 × 10** ^**−4**^	1.69 (1.28, 2.23)
**Positive family history of BD, %**	10.3	5.1	2.35 × 10^−2^	2.16 (1.09, 4.27)	10.9	4.6	**1.59 × 10** ^**−3**^	2.52 (1.40, 4.58)

The study of rs76546355 was performed in all BD patients (n = 973) while the HLA-B*51 study was conducted in the subset of patients allelotyped for HLA-B (n = 681, of which 454 patients were positive for B*51 and the remaining 227 cases were B*51-negative). Significant *P*-values after Bonferroni correction (*P* ≤4.17 × 10^−3^) are highlighted in bold. The odds ratio (OR) and 95% CI are indicated for nominally significant associations.

## Discussion

In this study, we confirmed in a large Iranian dataset that B*51 is the *HLA-B* allele most strongly associated with increased BD risk and we detected protective associations of HLA-B*35, B*52 and B*40 alleles, which remain significant after Bonferroni correction. Association of non-B*51 alleles (for example, B*27, B*35, B*44, B*52, B*56, B*57) has been described mostly in isolated and underpowered reports [2,20-23], and may result from small samples or Type I errors secondary to the very large difference in HLA-B*51 frequency between cases and controls. To remove that potential effect of B*51 on the remaining alleles, we studied the genetic makeup of BD patients not carrying the B*51 allele and found an association of the B*15 allele only. B*15 has previously been reported to constitute a risk factor for BD in the Moroccan population [1] and it would be interesting to test if this allele accounts for BD risk in populations where the B*51 prevalence is very low, such as some African and Middle Eastern populations [24,25]. The HLA-B*51 association with BD in Iranian has been described previously [26,27], but the sample sizes were smaller and the methods were different (for example, serotyping versus genotyping).

Although HLA-B*51 has been consistently associated across populations of various ethnicities in numerous case-control association studies (reviewed and meta-analyzed in de Menthon *et al*. [18]), there is limited understanding of the biological role of HLA-B*51 in the pathogenesis of BD. Whether B*51 plays a role by itself, or is in LD with another common genetic variant directly involved in susceptibility to BD, has also been a matter of intense debate. Among the top BD GWAS variants tested here, we found that the rs76546355 SNP (named rs116799036 in Hughes *et al*. [7]) is the MHC polymorphism most associated with BD in an Iranian dataset, at a similar level to the HLA-B*51 association. To the best of our knowledge, the rs76546355/rs116799036 association described here is the most robust association ever reported with BD (unadjusted *P*-value = 2.51 × 10^−69^). Interestingly, the allele frequencies of rs76546355/rs116799036 in Iranian patients and controls are very similar to the frequencies in the Turkish cohort in Hughes *et al*. [7]. Very subtle ancestry differences can be detected in our dataset [28] but, as observed previously, our findings of association are not likely to be confounded by hidden population stratification. rs76546355/rs116799036 is located in the promoter region of *HLA-B*, approximately 24 kb upstream of *HLA-B* and 18 kb upstream of *MICA*. Furthermore, pairwise conditional analysis between HLA-B*51 and rs76546355/rs116799036 in our dataset showed that both variants remained associated after adjusting for the effect of each other, although the strength of the association decreased from 8.34 × 10^−48^ ≤ *P* ≤1.78 × 10^−46^ to 1.78 × 10^−8^ ≤ *P* ≤1.24 × 10^−7^. These results are consistent with the similar frequencies observed in cases (0.388 and 0.391) and controls (0.126 and 0.121, respectively) but with the low LD between these variants in Iranian (*r*^2^ = 0.58), and support the notion that rs76546355/rs116799036 and HLA-B*51 are independent genetic risk factors for BD in Iranians. These findings contrast with those reported by Hughes *et al*. [7] who found that rs116799036/rs76546355 remained associated when conditioned on HLA-B*5101 in Turkish and Italian datasets combined (*P* = 7.83 × 10^−9^), but the HLA-B*5101 association with BD was completely abrogated by conditioning on rs116799036/rs76546355 (*P* = 1.59 × 10^−1^). The discrepancy between our results may derive from possible differences between ethnicities and their haplotypic structure in this genomic region, their originally much stronger association of rs116799036/rs76546355 when compared to that of HLA-B*5101 (*P* = 9.42 × 10^−50^ and *P* = 4.37 × 10^−28^, respectively, in the meta-analysis) and from the fact that their HLA alleles were imputed with a four-digit resolution and not effectively genotyped [7]. Even though several high density SNP studies were conducted for BD [4-7,19], the MHC region is extremely polymorphic, and therefore we cannot formally exclude the possibility that both HLA-B*51 and rs76546355/rs116799036 are in LD with an as yet unidentified causal variant that may be identified by deep sequencing. Therefore, further studies to investigate the independence of these two genetic variants and the existence of another causal variant are warranted.

We found that HLA-B*51 positivity is increased in patients with a positive pathergy test and a positive family history, but these associations did not withstand Bonferroni correction. Even though the family history was not tested in previous B*51 genotype-phenotype correlations in Iranian patients, these studies revealed a later age at onset [29] and more ocular manifestations and positive pathergy tests [30] in B*51+ patients. A recent meta-analysis from Maldini *et al*. [8] showed that HLA-B*51/B5 carriage predominates in males and is associated with moderately higher prevalence of genital ulcers, ocular and skin manifestations, and a decreased prevalence of gastrointestinal involvement. In their meta-analysis of 72 publications with very diverse study characteristics (for example, sample size, classification criteria, genotyping method), pathergy test was not found associated with the presence of B*51 and positive family history was not tested. Other studies have reported that the HLA-B5 positivity is much more frequent in familial BD than in sporadic cases [31]. Divergence between our nominally significant findings and those from the Maldini *et al*. [8] meta-analysis may result from between-study heterogeneity (for example, for eye involvement) or publication bias (for example, for eye involvement and male sex), but the lack of consistent associations in Iranian studies and across different populations reinforces the notion that the clinical and demographic characteristics of B51-positive and B51-negative patients are virtually indistinguishable. We also found that positivity for the risk allele at rs76546355/rs116799036 predominates in BD cases with genital ulcers, positive pathergy test and positive BD family history. Unfortunately, the association of two SNPs (rs4947296 and rs4959053) described as the most strongly associated with BD (1.80 × 10^−26^ ≤ *P* ≤4.01 × 10^−13^) in GWAS conducted in Japanese, Korean and Han Chinese [5,6,19] could not be assessed in this study.

## Conclusions

In conclusion, we found that the HLA-B*51 allele and the rs76546355/rs116799036 MHC SNP are independent genetic risk factors for BD in Iranians, and that positivity for the rs76546355/rs116799036 risk allele, but not for the B*51 allele, correlates with specific demographic characteristics or clinical manifestations in BD patients. In the near future, deep sequencing studies of the MHC locus in large datasets of patients and controls are expected to shed further light on the strongest BD genetic risk factors in this complex chromosomal region.

## Additional file

Additional file 1: Table S1. Primer sequences used to genotype the three single nucleotide polymorphisms (SNPs) investigated in this study.

## Abbreviations

AAE: age-at-examination
BD: Behçet’s disease
GWAS: genome-wide association study
HLA: human leukocyte antigen
HWE: Hardy-Weinberg equilibrium
LD: linkage disequilibrium
MHC: major histocompatibility complex
OR: odds ratio
SNP: single nucleotide polymorphism
