# Supplementary material for: Characterization of the major histocompatibility complex locus association with Behçet’s disease in Iran
Source: Arthritis Res Ther. 2015 Mar 19;17(1):81. doi: 10.1186/s13075-015-0585-6 (PMC4415285; doi:10.1186/s13075-015-0585-6)
Supplement: Additional file 1: Table S1. — Primer sequences used to genotype the three single nucleotide polymorphisms (SNPs) investigated in this study. [file 13075_2015_585_MOESM1_ESM.doc]

**Table S1 Sequences of primers used to genotype the three SNPs investigated in this study**

| **SNP** | **PCR primer 1 (5’ to 3’)** | **PCR primer 2 (5’ to 3’)** | **Extension primer (5’ to 3’)** |
| --- | --- | --- | --- |
| **rs9260997** | ACGTTGGATGTCCAGACCAATTCCTGTTTC | ACGTTGGATGACAAGACCTGTGGAAGGCTG | tgccGTTTAGGCAGAGCTTGCTTTAGAG |
| **rs76546355** | ACGTTGGATGTCCATGTGAATTTTCACTCC | ACGTTGGATGTTTGGATCTTCCTCACTTTC | GGACACCTAGGGCAAAT |
| **rs2848713** | ACGTTGGATGTGCAAGAGGTGGGTTATTCC | ACGTTGGATGAGAAGTGCATCACCCATCTG | ATTCCTCACCCTATTTCTTGAT |
